# Deployment-based lifetime optimization for linear wireless sensor networks considering both retransmission and discrete power control

**DOI:** 10.1371/journal.pone.0188519

**Published:** 2017-11-29

**Authors:** Ruiying Li, Wenting Ma, Ning Huang, Rui Kang

**Affiliations:** 1 School of Reliability and Systems Engineering, Beihang University, Beijing, China; 2 Science and Technology on Reliability and Environmental Engineering Laboratory, Beijing, China; Southwest University, CHINA

## Abstract

A sophisticated method for node deployment can efficiently reduce the energy consumption of a Wireless Sensor Network (WSN) and prolong the corresponding network lifetime. Pioneers have proposed many node deployment based lifetime optimization methods for WSNs, however, the retransmission mechanism and the discrete power control strategy, which are widely used in practice and have large effect on the network energy consumption, are often neglected and assumed as a continuous one, respectively, in the previous studies. In this paper, both retransmission and discrete power control are considered together, and a more realistic energy-consumption-based network lifetime model for linear WSNs is provided. Using this model, we then propose a generic deployment-based optimization model that maximizes network lifetime under coverage, connectivity and transmission rate success constraints. The more accurate lifetime evaluation conduces to a longer optimal network lifetime in the realistic situation. To illustrate the effectiveness of our method, both one-tiered and two-tiered uniformly and non-uniformly distributed linear WSNs are optimized in our case studies, and the comparisons between our optimal results and those based on relatively inaccurate lifetime evaluation show the advantage of our method when investigating WSN lifetime optimization problems.

## Introduction

**Fig 1:**
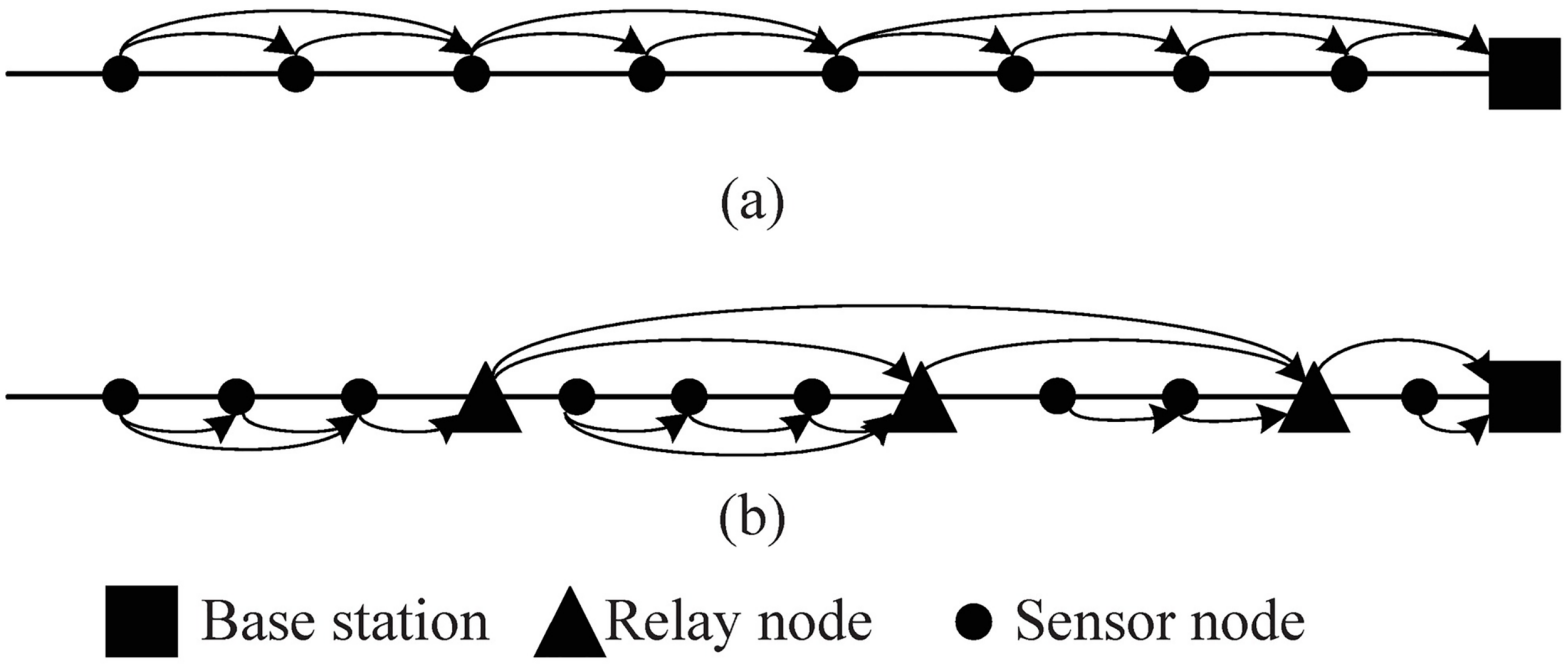
Typical linear WSNs. (a) one-tiered linear WSN; (b) two-tiered linear WSN.

As wireless communication and electronic miniaturization techniques have developed, wireless sensor networks (WSNs) are increasingly used in a wide variety of applications, such as industrial, military, business, habitat, health and environmental monitoring [1–7]. Many applications (e.g., monitoring oil, gas and water pipelines, railroads and subway tunnels, border surveillance, etc.) require placing sensors in a straight line. Such deployments are termed “linear WSNs” [8]. Usually, such WSNs include three types of nodes: (1) sensor nodes (SN), which can monitor the surrounding information, such as temperature, humidity, pressure and vibration, and transfer them as needed; (2) relay nodes (RN), which collect data from SNs, and deliver them to the base station; and (3) base station (BS) nodes, also called sink nodes, which collect data for further analysis. From a hierarchical point of view, linear WSNs can be classified into two categories: (1) one-tiered linear WSNs, which consist of only SNs and BSs, where the SNs transfer sensed information to the BS based on a routing protocol in hop by hop fashion; and (2) two-tiered linear WSN, which include all three of the node types described above, in which SNs collect information and transmit it to their parent RN, and the RNs forward the data to the BS (see Fig 1).

Because sensor and relay node batteries usually have limited capacity and are difficult to replenish or replace, the lifetime of a WSN is largely determined by its energy consumption [9–12]. Pioneers provided lots of methods to reduce the energy consumption, for example, energy-efficient data routing [13–16], data aggregation [17], effective node deployment and topology control [18], duty cycling, etc. Among such methods, the deployment based lifetime optimization attracts a lot of researchers. It has been reported that data communication (which involves both data transmission and reception) consumes the maximum energy in the life cycle of WSNs [1]. According to Stemm and Katz [19], the longer the transmission distance is, the more energy will be consumed. Therefore, pioneers focused on finding a node deployment that maximizes network lifetime [20, 21], Yao et al. [22] proposed a source location privacy protection strategy to ensure the security of the network. Several deployment strategies have been proposed to achieve load balance and avoid energy holes [23, 24]. For linear WSNs, Cheng et al. [25] proposed two greedy sensor placement and data transmission schemes for both linear and planar WSNs that balance the average energy consumption of each sensor to maximize the network lifetime or minimize the network cost. Chen et al. [26] provided a sensor deployment optimization problem for one-tiered linear WSNs that maximized network lifetime per cost using coverage and energy balance constraints. This problem is solved using a two-step solution: (1) optimize the sensor placement using a greedy strategy similar to [25], and (2) use numerical approximation to determine the optimal number of sensors. Ganesan et al. [27] studied the joint optimization of sensor placement and transmission structure that minimized energy consumption under a given number of nodes and a tolerable distortion rate. Liu and Mohapatra [28] studied how to optimally deploy back-haul nodes (i.e., RNs) for a two-tiered linear WSN. They provided a greedy deployment scheme that used the maximum coverage distance. Cao et al. [29], addressing one-tiered linear WSNs, developed a Lagrange-multiplier-based energy-efficient node placement scheme that balanced per-node energy consumption to maximize network lifetime. Hossain et al. [30] analyzed energy consumption in linear wireless camera sensor networks and proposed a node placement scheme that yielded equal energy dissipation over the network. Although the optimization models in [25, 29] are for one-tiered linear WSNs, the authors also declared their methods suitable for two-tiered linear WSNs, in which the node deployment can be optimized in two tiers. Only RN deployment—including the number of RNs and the node distances between them—is optimized for the higher tier. In the lower tier, each cluster is regarded as a separate sensor network in which RNs are regarded as sink nodes. SN deployment can be optimized in the same manner. Additional linear WSN lifetime optimization studies can be found in [31–33].

Considering that WSNs are usually deployed in harsh environments, data transmission is often unreliable; therefore, retransmission is commonly applied to improve the transmission success rate. She et al. [34] proposed an analytical method to quantify the energy consumption of hop-by-hop and end-to-end retransmission schemes, demonstrating that hop-by-hop retransmission consumes less energy under different bit-error-rates. Li et al. [35, 36] proposed deployment-based lifetime optimization models for one-tiered linear and flat WSNs under hop-by-hop retransmission. Their results illustrated that optimal node deployments with and without retransmission are quite different; therefore, retransmission should be considered when studying deployment-based lifetime optimization problems.

On the other hand, the power of nodes in WSN are usually limited to a limited number of values in practical settings, and cannot be infinitely tunable [37–40]. For example, the Mica2 [41], Mica3 [42], MicaZ [43], and Tmote Sky Sensor [44] all have discrete power levels. However, in most works, linear deployment-based optimization models are built based on the widely used continuous power model proposed by Heinzelman et al. [45], in which both the free space (power loss) and multipath fading (power loss) channel models were considered. This ideal power model facilitates deployment-based optimization but may lead to misleading results. Aslam et al. [46] and Banerjee et al. [47] compared the continuous and discrete power models available from sensor manufacturers in terms of energy consumption and lifetime calculations, and the results show large differences. Guo et al. [48] studied the lifetime optimization problem for a one-tiered linear WSN used to monitor oil pipelines and proposed two efficient placement heuristics that minimized the energy consumption using the realistic discrete power model. They found that the optimal sensor deployment scheme obtained when using the discrete power model was different from the one they obtained using the ideal power model; therefore, the realistic power model should also be considered when studying WSN deployments.

Table 1 compares these deployment-based optimization approaches for linear WSNs. None of these approaches considers both realistic discrete power model and retransmission model deployments. Consequently, in this paper, we combine the two models in the deployment-based lifetime optimization for linear WSNs and focus on how they influence the optimal deployment. To the best of our knowledge, our lifetime model and deployment-based lifetime optimization model for linear WSNs are among the first generic models to consider retransmission and discrete power level. Together, these provide a more accurate estimation of network lifetime and better node deployment.

**Table 1:** A comparison between different node deployment approaches for linear WSNs.

Paper	Deployment	Tiers	Node type	Time	Objective	Constraints	Energy model	Retransmission
[25]	Deterministic, non-uniform	One	SN	Periodically	Lifetime^(1)^ or cost	Coverage, number of nodes, flow	Continuous	Neglected
[26]	Deterministic, non-uniform	One	SN	Event driven	Lifetime per unit cost^(2)^	Coverage, energy balance	Continuous	Neglected
[27]	Deterministic, non-uniform	One	SN	Periodically	Total energy consumed	Coverage, connectivity, number of nodes, specified distortion bounds	Continuous	Neglected
[28]	Deterministic, non-uniform	Two	RN	Periodically	Length of monitoring area	Lifetime^(1)^, number of RNs, coverage	Continuous	Neglected
[29]	Deterministic, non-uniform	One	SN	Arbitrary	Lifetime^(3)^	Length of monitoring area, connectivity, cost	Continuous	Neglected
[30]	Deterministic, non-uniform	One	SN	Periodically	Energy balance	Length of monitoring area, number of SNs	Continuous	Neglected
[31]	Deterministic, uniform	One	BS	Periodically	Number of BSs	Number of SNs, connectivity	Continuous	Neglected
[32]	Deterministic, uniform and non-uniform	One	SN	Arbitrary	Total energy consumed	Number of nodes, length of monitoring area	Continuous	Neglected
[33]	Deterministic and random, non-uniform	One	SN	Periodically	Energy balance	Number of SNs, length of monitoring area	Continuous	Neglected
[36]	Deterministic, uniform and non-uniform	One	SN	Periodically	Lifetime^(1)^	Coverage, connectivity, transmission success rate, number of SNs	Continuous	Considered
[48]	Deterministic, uniform and non-uniform	One	SN	Periodically	Lifetime^(1)^	Number of SNs, length of monitoring area, connectivity	Discrete	Neglected

Notes:

^(1)^ the period of time from network initialization until the first sensor death occurs;

^(2)^ the network lifetime (see 1) divided by the number of sensors deployed in the network; and

^(3)^ the initial energy divided by the average energy consumption per node.

## Methods

### Problem description

This paper aims at combining retransmission and discrete power control in the deployment based lifetime optimization for linear WSNs. To clearly discuss such problem, the node deployment, data sensing and transmission, retransmission, discrete power control are described as follows for further analysis.

#### Node deployment

**Fig 2:**
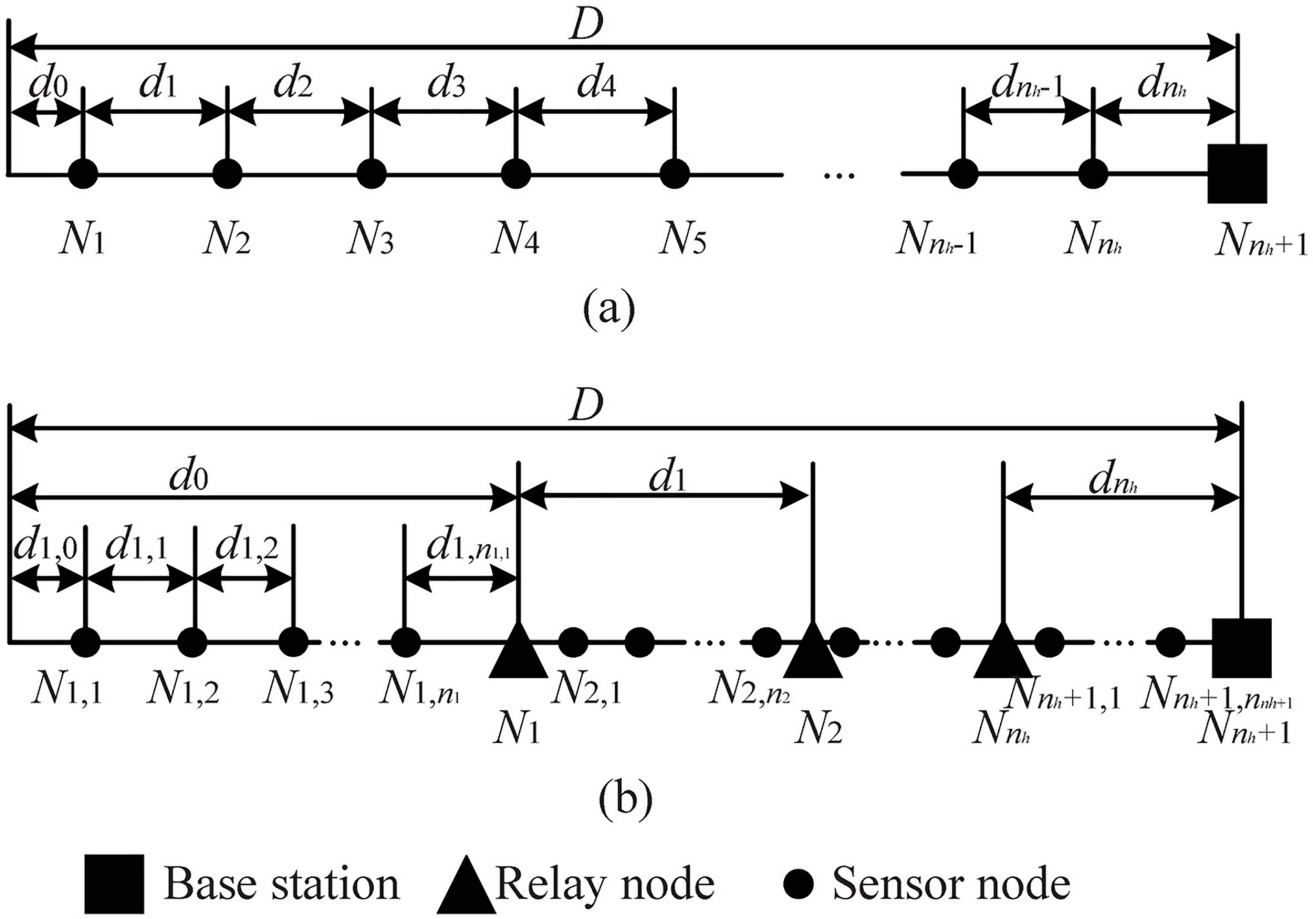
Linear WSN topologies. (a) one-tiered linear WSN; (b) two-tiered linear WSN.

Fig 2 shows typical one-tiered and two-tiered linear WSNs, where all nodes are arrayed in lines as a structured network to monitor a linear space with length *D*. We consider that the network includes only one BS located at one end of the line; if the BS is located between nodes, the network can be split into two independent deployment-based optimizations, one each for nodes on either side of the links with the BS at one end. Let *n*_*h*_ and *n*_*l*_ denote the numbers of nodes in the higher and lower tiers, respectively. For a one-tiered WSN, there is no hierarchy, and we denote SNs as nodes in the higher tier. For the two-tiered WSN, RNs are in the higher tier, and SNs are in the lower tier.

In Fig 2, *n*_*h*_ nodes located in the higher tier are denoted as $N_{1},N_{2},\ldots ,N_{n_{h}}$, and the BS is denoted as $N_{n_{h}+1}$. For the two-tiered WSN, the SNs in the lower tier have a parent node (i.e, the neighboring RN closer to the BS or the BS itself, where the BS is denoted as $N_{n_{h}+1}$), and their sensing data will be transmitted to the parent node for further forwarding. Each parent node *N*_*i*_ serves *n*_*i*_ child SNs denoted as $N_{i,1},N_{i,2},\ldots ,N_{i,n_{i}}$; therefore, the number of nodes in the lower layer can be calculated as $n_{l}=\sum_{i=1}^{n_{h}+1}n_{i}$. Let *d*_*i*_ and *d*_*i*,*j*_ denote the distances from Node *N*_*i*_ to its next hop in the higher layer and from Node *N*_*i*,*j*_ to its next hop in the lower layer, respectively.

#### Data sensing and transmission

In WSNs such as those in Fig 2, the SNs are responsible for sensing, and each SN has a sensing range of *R*_*s*_. Each node in the WSNs has the ability to transmit and relay data according to the routing protocol. Nodes in the higher and lower layers have communication ranges of *R*_*t*,*h*_ and *R*_*t*,*l*_, respectively. In our paper, we assume that data transmission follows the shortest path routing. In the higher layer, Node *N*_*i*_ can transmit the sensing data only to Node *N*_*i*+1_. In the lower layer, Node *N*_*i*,*j*_ can transmit the sensing data only to Node *N*_*i*,*j*+1_ (the parent node *N*_*i*_ can also be denoted as $N_{i,n_{i}+1}$). The data transmission mode can also be seen in Fig 1.

#### Retransmission

According to the packet reception rate model of Zuniga and Krishnamachari [49], the retransmission rate can be obtained according to the sensor modulation, encoding scheme and path loss models. Consider Mica2 (with non-coherent FSK modulation and encoded by Manchester code) as an example. Using the one slope log-distance path loss model from [49], the retransmission rate for MICA2 motes can be obtained as follows:

$$\begin{array}{l} \begin{array}{l} RR\left(d,m\right)=1-{\left[1-\frac{1}{2}e^{\frac{\left(P_{o}-PL\left(d\right)-P_{n}\right)B_{N}}{2r}}\right]}^{2m-l}\\ =1-{\left[1-\frac{1}{2}e^{\frac{\left(P_{o}-PL\left(d_{0}\right)-10\alpha log_{10}\frac{d}{d_{0}}-X_{\delta }-P_{n}\right)B_{N}}{2r}}\right]}^{2m-l}, \end{array} \end{array}$$ (1)

where *PL*(*d*) is the path loss model, *d* is the transmission distance, *P*_*n*_ is the noise floor (in dB), *B*_*N*_ is the noise bandwidth, *r* is the data transmission rate (bit/sec), *PL*(*d*_0_) is the path loss at the reference distance *d*_0_, *α* is the path loss exponent (2 ≤ *α* ≤ 4) depending on different channel models, *X*_*δ*_ is a lognormal variable with standard deviation *δ* (in dB), and *m* and *l* are the lengths of the packet and preamble (in bits), respectively.

#### Discrete power control

Node datasheets provide a data source for the discrete power levels. For example, the datasheet [41] of Mica2 which uses CC1000 chips provides 26 different transmission power levels and their corresponding consumption. The transmission power consumption at different power levels can be computed using the current consumption multiplied by the typical voltage. Table 2 presents the transmission power consumption for various Mica2 output power levels.

**Table 2:** Transmission power and range for Mica2 (*f* = 868 MHz, *α* = 3.95).

*P*_*o*_ (dBm)	*P*_*t*_ (mW)	*R*_max_ (m)	*P*_*o*_ (dBm)	*P*_*t*_ (mW)	*R*_max_ (m)	*P*_*o*_ (dBm)	*P*_*t*_ (mW)	*R*_max_ (m)
-20	25.8	19.30	-11	29.7	32.62	-2	45.3	55.13
-19	26.4	20.46	-10	30.3	34.58	-1	47.4	58.44
-18	27.0	21.69	-9	31.2	36.66	+0	50.4	61.95
-17	27.0	22.99	-8	31.8	38.86	+1	51.6	65.67
-16	27.3	24.38	-7	32.4	41.19	+2	55.5	69.61
-15	27.9	25.84	-6	33.3	43.67	+3	57.6	73.79
-14	27.9	27.39	-5	41.4	46.29	+4	63.9	78.22
-13	28.5	29.03	-4	43.5	49.07	+5	76.2	82.92
-12	29.1	30.74	-3	43.5	52.01			

Note: *f* is the frequency, *P*_*o*_ is output power, *P*_*t*_ is transmission power consumption, and *R*_max_ is the maximum transmission range at such power level.

### Optimization model

Considering both retransmission and discrete power consumption, this section presents an optimization model to find the optimal node deployment that maximizing the network lifetime, subject to coverage, connectivity, and transmission success rate constraints as well as the maximum allowable number of nodes and target length. The node deployment scheme includes both the number of nodes placed in the linear sensor network and the distance between nodes. Both objective and constrains are discussed as follows.

### Objective: Maximizing the network lifetime

In our paper, we assume that data are being sensed and transmitted periodically in the WSN, and that no nodes will fail until their energy is exhausted. This assumption is reasonable, because WSN lifetimes range from only hundreds of days to several years due to their limited energy capacity, but node mean time between failures (MTBF) is calculated in tens of years. Therefore, energy consumption largely determines the lifetime of WSNs, and we adopt the common definition of lifetime as the duration between the time network begins operating and the time when the first node has exhausted all its energy and dies [50], which is equivalent to the minimum node lifetime. This lifetime definition is now widely used in WSN lifetime optimization research, because network lifetime is optimized when the energy consumptions of different sensors are nearly balanced. In other words, when the first node dies, the energy of other nodes will also run out very soon. We suppose that the capacity of the BS is unlimited; therefore, we have the following:

$$\begin{array}{c} L=\min_{\text{all}\;i,j}(L_{i},L_{i,j})=\min_{\text{all}\;i,j}[\frac{E_{h}\left(0\right)}{E_{i}},\frac{E_{l}\left(0\right)}{E_{i,j}}]t, \end{array}$$ (2)

where *L*_*i*_ and *L*_*i*,*j*_ are the lifetime of Node *N*_*i*_ in the higher layer (1 ≤ *i* ≤ *n*_*h*_) and Node *N*_*i*,*j*_ (1 ≤ *i* ≤ *n*_*h*_ + 1, 1 ≤ *j* ≤ *n*_*i*_) in the lower layer, respectively. These nodes are powered by non-rechargeable batteries with an initial energy of *E*_*h*∣*l*_(0), which will be consumed as *E*_*i*_ and *E*_*i*,*j*_ during a data-gathering cycle *t*.

**Fig 3:**
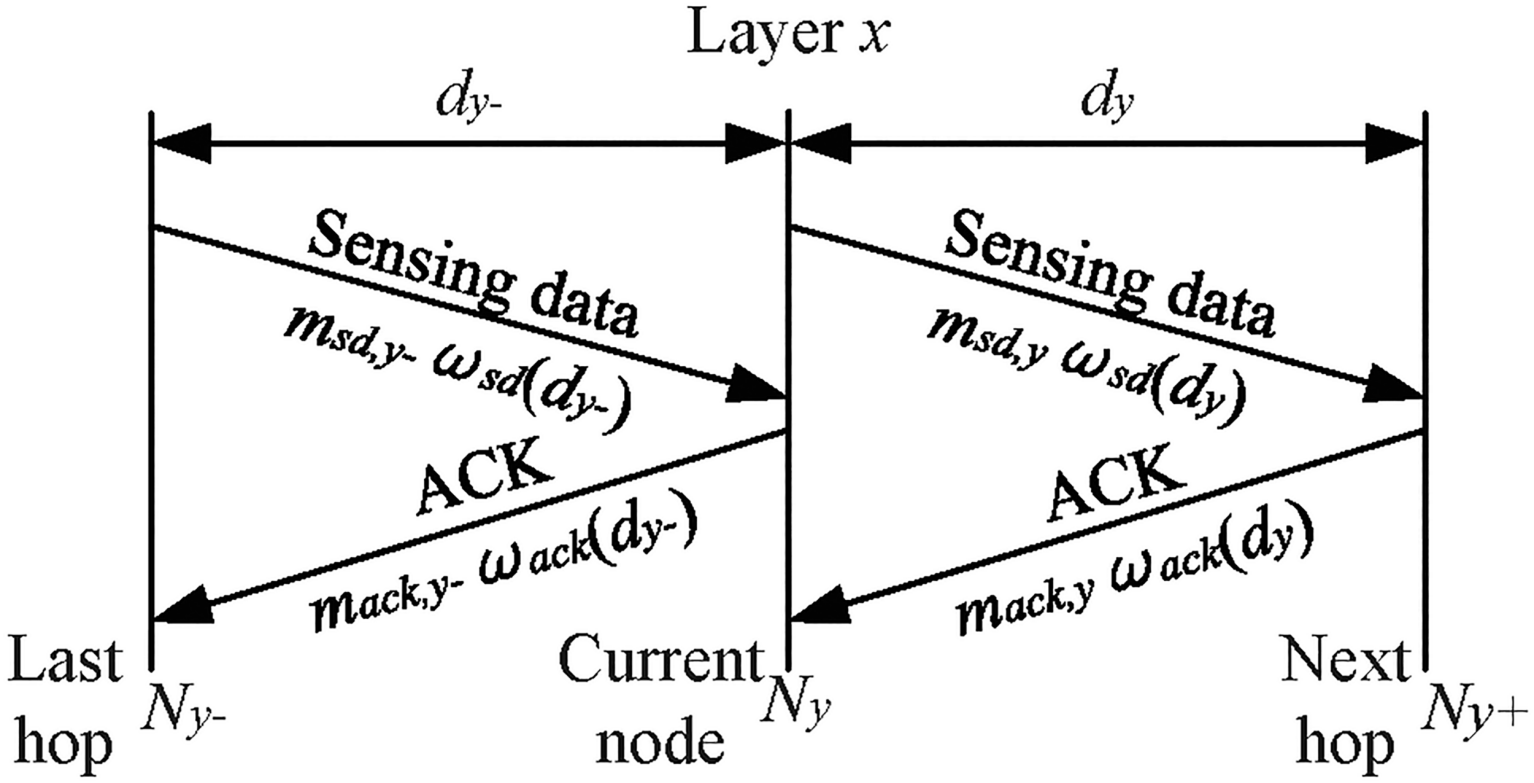
Data transmitted and received at Node *N*_*y*_. Note: $N_{y^{+}}$ and $N_{y^{-}}$ represent the next hop and the last hop of Node *N*_*y*_, respectively.

Generally, there are three main ways for energy consumption: data transmission, data reception and idle [19]. The energy consumed during data sensing or sleep are ignored. This approach is consistent with node datasheets [41–43], in which one can see that the energy consumption in power-down mode and during sensing is negligible compared to the energy consumption during transmit, receive and idle modes. During transmission and reception, there are two types of data, sensing data and control data, and retransmissions caused by harsh environments should also be considered. The data amount depends on the data sensed during the sensing period, the routing protocol and the MAC protocol. For example, if the CSMA/CA protocol is used without RTS/CTS, two types of data are transmitted (sensing data and acknowledgement data). After the sensing data is transmitted, if it is received successfully, the receiver sends an ACK response back to the transmitter; otherwise, the transmitter will retransmit the sensing data until a maximum retry threshold is reached. The data transmitted and received by Node *N*_*y*_ can be seen in Fig 3.

Using the discrete power model, the energy consumed for data transmission, reception and idle at Node *N*_*y*_ of Layer *x* during a data-gathering cycle *t* can be calculated as follows:

$$\begin{array}{c} \{\begin{array}{c} E_{t,y}=P_{t,x}\left(d_{y}\right)t_{t,sd}+P_{t,x}\left(d_{y^{-}}\right)t_{t,ack},\\ E_{r,y}=P_{r,x}\left(t_{r,sd,y}+t_{r,ack}\right),\\ E_{id,y}=P_{id,x}\left(t-t_{t,sd}-t_{t,ack}-t_{r,sd}-t_{r,ack}\right), \end{array} \end{array}$$ (3)

where *x* represents ‘*h*∣*l*′ in the higher or lower layer, *y* represents node *i* in the higher layer or node *i*, *j* in the lower layer; $P_{t,x}(d_{y}|d_{y^{-}})$ is the power consumed by nodes in layer *x* to transmit data over a certain distance *d*_*y*_ or $d_{y^{-}}$(the transmission power consumption at different power levels can be obtained from the sensor datasheet shown in Table 2); *P*_*r*,*x*_ and *P*_*id*,*x*_ are the power consumed at data receiving state and idle state for nodes of Layer *x*, respectively, and are both constants obtainable from the node datasheet; *t*_*t*∣*r*,*sd*∣*ack*_ is the time duration of sensing data or ACK transmitted from or received by Node *N*_*y*_ in a data gathering cycle *t*, they can be calculated as

$$\begin{array}{c} \{\begin{array}{c} t_{t,sd}=\frac{m_{sd,y}\omega_{sd}\left(d_{y}\right)}{r_{x}},\\ t_{r,sd}=\frac{m_{sd,y^{-}}\omega_{sd}\left(d_{y^{-}}\right)}{r_{x}},\\ t_{t,ack}=\frac{m_{ack,y^{-}}\omega_{ack}\left(d_{y^{-}}\right)}{r_{x}},\\ t_{r,ack}=\frac{m_{ack,y}\omega_{ack}\left(d_{y}\right)}{r_{x}}, \end{array} \end{array}$$ (4)

where $m_{sd|ack,y|y^{-}}$ and $\omega_{sd|ack}(d_{y|y^{-}})$ are the amount and the expected number of transmission attempts for sensing data or ACK needed to transmit between Nodes *N*_*y*_ and $N_{y^{+}}$ with distance *d*_*y*_ or between Nodes $N_{y^{-}}$ and *N*_*y*_ with distance $d_{y^{-}}$(see Fig 3), and *r*_*x*_ is the data processing rate of the nodes in Layer *x*. For RNs, note that two types of sensing data reception and ACK transmission (i.e., the data transmission from the last RN or SN to that RN and the ACK response) exist. The expected numbers of transmission attempts between two hops can be obtained by enumerating all the possibilities shown in S1 Appendix.

According to Eq (3), the energy consumption at Node *N*_*y*_ of Layer *x* during a data-gathering cycle can be written as

$$\begin{array}{c} E_{y}=E_{t,y}+E_{r,y}+E_{id,y}. \end{array}$$ (5)

#### Constraint 1: Coverage

The coverage of a linear WSN can be measured by the coverage percentage, which is the ratio of the length covered by the sensors to the target length. The coverage of the WSN is determined by the sensing range of the sensors and the distance between nodes. The segments between two sensors are covered by the sensors at both ends, while those segments at both ends of the linear WSN are covered by only one sensor. For one-tiered linear WSN, the coverage length of the segment between Node *N*_*i*_ and Node *N*_*i*+1_ (the BS at one end is regarded as Node *N*_*h*+1_, and the other end is regarded as Virtual Node *N*_0_) can be computed by

$$\begin{array}{c} CovL_{i}=\{\begin{array}{c} \min(d_{i},R_{S}),\;\;\left(i=0\;\text{or}\;n_{h}\right),\\ \min(d_{i},2R_{S}),\;\left(1\leq i<n_{h}\right). \end{array} \end{array}$$ (6)

For a two-tiered linear WSN, the coverage length of the segment between Node *N*_*i*,*j*_ and the next SN (the BS at one end is regarded as the next SN of Node $N_{n_{h}+1,n_{n_{h}+1}}$, and the other end is regarded as Node *N*_1,0_) can be computed by

$$CovL_{i,j}=\{\begin{array}{ll} \text{min}\left\{d_{i,j},R_{S}\right\}, & \left(i=1,j=0\;\text{or}\;i=n_{h}+1,j=n_{n_{h}+1}\right),\\ \text{min}\left\{d_{i,n_{i}}+d_{i+1,0},2R_{S}\right\}, & \left(1\leq i\leq n_{h}\right),\\ \text{min}\left\{d_{i,j},2R_{S}\right\}, & \left(\text{others}\right). \end{array}$$ (7)

Dividing the sum of the coverage lengths for all segments by the target length *D*, the coverage percentage can be obtained. The coverage percentage constraint for one-tiered and two tiered linear WSNs can be denoted as follows:

$$\begin{array}{c} \{\begin{array}{c} Cov_{1wsn}=\frac{\sum_{i=0}^{n_{h}}CovL_{i}}{D}\geq Cov_{wsn}^{*},\\ Cov_{2wsn}=\frac{CovL_{1,0}+\sum_{i=1}^{n_{h}+1}\sum_{j=1}^{n_{i}}CovL_{i,j}}{D}\geq Cov_{wsn}^{*}, \end{array} \end{array}$$ (8)

where the numerator and denominator of the equations are the covered length and the target length, respectively, and $Cov_{wsn}^{*}$ is the coverage percentage constraint.

Specifically, if $Cov_{wsn}^{*}=1$, Eq (8) can be simplified to

$$\{\begin{array}{ll} d_{i}\leq R_{S}, & \left(i=0\;\text{or}\;i=n_{h}\right),\\ d_{i}\leq 2R_{S}, & \left(1\leq i<n_{h}\right) \end{array}$$ (9)

and

$$\begin{array}{c} \{\begin{array}{c} d_{i,j}\leq R_{S},\;\left(i=1,j=0\;\text{or}\;i=n_{h}+1,j=n_{n_{h}+1}\right),\\ d_{i,n_{i}}+d_{i+1,0}\leq 2R_{S},\;\left(1\leq i\leq n_{h}\right),\\ d_{i,j}\leq 2R_{S},\;\left(\text{others}\right), \end{array} \end{array}$$ (10)

for one-tiered and two-tiered linear WSNs, respectively. In this paper, the coverage is defined as a percentage. For other coverage measures, such as k-coverage, we refer readers to [51] and [52] for details.

#### Constraint 2: Connectivity

Similar to coverage, WSN connectivity can be measured as the connectivity percentage, which is the number of connected SNs divided by their total number. The connectivity of a linear WSN depends on the transmission range of the nodes and the distance between hops. The connectivity between Node *N*_*y*_ and its next hop is defined as

$$\Psi_{y}=\{\begin{array}{ll} 1, & \text{if}\;d_{y}\leq R_{t,x},\\ 0, & \text{others}, \end{array}$$ (11)

where *d*_*y*_ is the distance between Node *N*_*y*_ and its next hop (see Fig 3).

As the data are transmitted hop by hop, if data on Node *N*_*y*_ cannot transmit to its next hop, i.e., *d*_*y*_ > *R*_*t*,*x*_, all sensors further away than *N*_*y*_ cannot be connected with the BS and vice versa. For one-tiered or two-tiered linear WSNs, the connectivity constraint of the whole network can be calculated as

$$\begin{array}{c} \{\begin{array}{c} Con_{1wsn}=\frac{\overset{n_{h}}{\underset{i=1}{\text{max}}}\left[\left(n_{h}-i+1\right)\prod_{k=i}^{n_{h}}\Psi_{k}\right]}{n_{h}}\geq Con_{wsn}^{*},\\ Con_{2wsn}=\frac{Con_{n_{h}+1}+\overset{n_{h}}{\underset{i=1}{\text{max}}}\left(\sum_{k=i}^{n_{h}}Con_{k}\prod_{k=i}^{n_{h}}\Psi_{k}\right)}{n_{l}}\geq Con_{wsn}^{*}, \end{array} \end{array}$$ (12)

where the numerators are the number of connected SNs, and *Con*_*i*_ is the number of SNs that can be connected to a parent node *N*_*i*_ in the two tiered WSN, and can be calculated as

$$\begin{array}{c} Con_{i}=\max_{j=1}^{n_{i}}[(n_{i}-j+1)\prod_{k=j}^{n_{i}}\Psi_{i,k}], \end{array}$$ (13)

for 1 ≤ *i* ≤ *n*_*h*_ + 1.

In particular, if $Con_{wsn}^{*}=1$, Eq (12) can be simplified to

$$\begin{array}{c} d_{i}\leq R_{t,h},\;\left(1\leq i\leq n_{h}\right) \end{array}$$ (14)

and

$$\{\begin{array}{ll} d_{i,j}\leq R_{t,l}, & \left(1\leq i\leq n_{h}+1\;\text{and}\;1\leq j\leq n_{i}\right),\\ d_{i}\leq R_{t,h}, & \left(1\leq i\leq n_{h}\right), \end{array}$$ (15)

for one-tiered and two-tiered linear WSNs, respectively.

#### Constraint 3: Transmission success probability

In harsh environments, not all data sent from connected sensors can be transmitted to the sink node successfully, and the transmission success probability is the amount of sensing data successfully transmitted to BS divided by the total amount of sensed data. According to the retransmission model and the communication protocol, we can calculate the transmission success probability between any two adjacent hops by enumerating and adding all the transmission success probabilities together. See S1 Appendix for details.

Let *S*_*i*_ and *S*_*i*,*j*_ denote the transmission success probability from Node *N*_*i*_ to its next hop in the higher layer and from Node *N*_*i*,*j*_ to its next hop in the lower layer, respectively. In a one-tiered linear WSN, the transmission success probability from Node *N*_*i*_ to the BS can be computed as $\prod_{k=i}^{n_{h}}S_{k}$. While the success probability from SN *N*_*i*,*j*_ to its parent node *N*_*i*_ can be calculated as $\prod_{k=j}^{n_{i}}S_{i,k}$ for the two-tiered linear WSN. The transmission success probability constraint for the entire WSN can be denoted as follows:

$$\{\begin{array}{ll} Suc_{1wsn} & =\frac{\sum_{i=1}^{n_{h}}\left(\prod_{k=i}^{n_{h}}S_{k}\times m_{sd,i}\right)}{\sum_{i=1}^{n_{h}}m_{sd,i}}\geq Suc_{wsn}^{*},\\ Suc_{2wsn} & =\frac{Suc_{n_{h}+1}+\sum_{i=1}^{n_{h}}\left(\prod_{k=i}^{n_{h}}S_{k}\times Suc_{i}\right)}{\sum_{i=1}^{n_{h}+1}\sum_{j=1}^{n_{i}}m_{sd,i,j}}\geq Suc_{wsn}^{*}. \end{array}$$ (16)

for one-tiered and two-tiered linear WSNs, respectively. Here, *m*_*sd*,*i*_ is the data sensed by Node *N*_*i*_, $Suc_{wsn}^{*}$ is the connectivity constraint, *Suc*_*i*_ is the amount of sensing data that can be successfully transmitted to node *N*_*i*_ in the two tiered WSN, and can be calculated as

$$\begin{array}{c} Suc_{i}=\sum_{j=1}^{n_{i}}(\prod_{k=j}^{n_{i}}S_{i,k}\times m_{sd,i,j}), \end{array}$$ (17)

for 1 ≤ *i* ≤ *n*_*h*_ + 1, where *m*_*sd*,*i*,*j*_ is the data sensed by SN *N*_*i*,*j*_.

#### Constraint 4: The maximum allowable number of nodes

In our optimization problem, the constraint of the number of nodes can be denoted as follows:

$$\{\begin{array}{l} n_{h}\leq n_{h}^{*},\\ n_{l}\leq n_{l}^{*}, \end{array}$$ (18)

where $n_{h|l}^{*}$ is the maximum allowable number of nodes in the higher or lower layer.

#### Constraint 5: The target length

For a linear WSN used to monitor a linear space with length *D*, the constraint of the target length can be denoted as

$$\begin{array}{c} \sum_{i=0}^{n_{h}}d_{i}=D. \end{array}$$ (19)

and

$$\{\begin{array}{l} \sum_{j=0}^{n_{i}}d_{i,j}=d_{i-1},\;\text{for}\;1\leq i\leq n_{h}+1,\\ \sum_{i=0}^{n_{h}}d_{i}=\sum_{i=1}^{n_{h}+1}\sum_{j=0}^{n_{i}}d_{i,j}=D. \end{array}$$ (20)

for one-tiered and two-tiered WSNs, respectively.

#### Optimization model

Using the above models, we can achieve our optimization model by maximizing Eq (2) and setting the above constraints as Eqs (8), (12), (16), (18) and (19) (or (20)). This optimization model aims to find the optimal node deployment, including the number of different types of nodes and the distance between nodes.

## Results and discussion

In this section, we present the use of our optimization model to find an optimal node deployment scheme that maximizes network lifetime. To illustrate the effectiveness of our method, the optimal results obtained through our model are compared with the ones using previous models.

### One-tiered example

In our example, MICA2 motes each with a CC1000 chip are used as the SNs. We assume that each sensor can obtain the same amount of sensing data (*m*_*sd*_) during a data-gathering cycle *t*, and transmit it to the BS using the shortest path routing algorithm (i.e., SN *N*_1_ sends its data to its neighbor *N*_2_, and *N*_2_ sends its own data as well as the relayed data of *N*_1_ to node *N*_3_, and so on). The transmission power control scheme reported in [53] is adopted (see Table 2), and different transmission power levels result in different maximum transmission ranges. The MAC protocol adopts the CSMA/CA protocol without RTS/CTS, and the maximum number of retry threshold is specified to be 2 for any one sensing data transmission. To save energy, the zero-idle protocol described in [54] is adopted. Other parameters of our one-tiered linear WSN are shown in Tables 3 and 4, where the latter shows values for SNs in the higher level.

**Table 3:** Parameters for the WSN.

Parameters	Value	Parameters	Value	Parameters	Value
*D*	1000 m	*L*_*sd*_	160 bits	$Cov_{wsn}^{*}$	1
*m*_*sd*_	448 bits	*L*_*ack*_	120 bits	$Con_{wsn}^{*}$	1
*m*_*ack*_	120 bits	*t*	300 s	$Suc_{wsn}^{*}$	0.9

**Table 4:** Parameters for the SNs.

Parameters	Value	Parameters	Value	Parameters	Value
*R*_*s*_	50 m	*α*	3.95	*PL*(1)	31 dB
*R*_*t*_	82.92 m	*P*_*r*_	35.4 mW	*β*_1_	1.53 *μ*J/bit
*E*(0)	5400 J	*P*_*id*_	35.4 mW	*β*_2_	0.0743 pJ/bit/m^3.95^
*r*	19.2 kbit/s	*B*_*N*_	30 kHz	*β*_3_	1.84 *μ*J/bit
n*	50	*P*_*n*_	-111 dB	*β*_4_	1.84 *μ*J/bit

Here, the values of *β*_1_, *β*_2_, *β*_3_ and *β*_4_ are provided for comparison with the continuous power model proposed by [45], see S2 Appendix for details. To ensure basic data consistency between the continuous and discrete power models, the values of these parameters are modified using curve fitting with 95% confidence bounds.

In the linear WSN deployment-based lifetime optimization problem, there are two typical situations, i.e., WSNs with sensors uniformly and non-uniformly distributed along the line [32, 36, 48]. In this section, we mainly discuss non-uniformly distributed WSNs, which is used to solve the “energy hole” problem [25]. The uniformly situation can be regarded as a similar problem with an extra constraint $d_{i}=\frac{D-R_{s}}{n_{h}}$. The optimization model can be written as follows:

$$\begin{array}{c} \max\;L=\min_{i=1}^{n_{h}}\frac{E_{h}\left(0\right)}{E_{i}}t, \end{array}$$ (21)

subject to

$$\begin{array}{c} \begin{array}{c} \frac{\sum_{i=0}^{n_{h}}CovL_{i}}{D}\geq Cov_{wsn}^{*},\\ \frac{\max_{i=1}^{n_{h}}[(n_{h}-i+1)\prod_{k=i}^{n_{h}}\Psi_{k}]}{n_{h}}\geq Con_{wsn}^{*},\\ \frac{\sum_{i=1}^{n_{h}}(\prod_{k=1}^{n_{h}}S_{k}\times m_{sd,i})}{\sum_{i=1}^{n_{h}}m_{sd,i}}\geq Suc_{wsn}^{*},\\ n_{h}\leq n_{h}^{*},\\ \sum_{i=0}^{n_{h}}d_{i}=D. \end{array} \end{array}$$ (22)

Specifically, when $Cov_{wsn}^{*}=1$, $Con_{wsn}^{*}=1$ and all *m*_*sd*,*i*_ = *m*_*sd*_, Eq (22) can be simplified to

$$\begin{array}{c} \begin{array}{c} d_{0}\leq R_{s},\\ d_{n_{h}}\leq \min(R_{s},R_{t}),\\ d_{i}\leq \min(2R_{s},R_{t}),\text{for}\;1\leq i<d_{n_{h}},\\ \frac{\sum_{i=1}^{n_{h}}(\prod_{k=1}^{n_{h}}S_{k})}{n_{h}}\geq Suc_{wsn}^{*},\\ n_{h}\leq n_{h}^{*},\\ \sum_{i=0}^{n_{h}}d_{i}=D. \end{array} \end{array}$$ (23)

**Fig 4:**
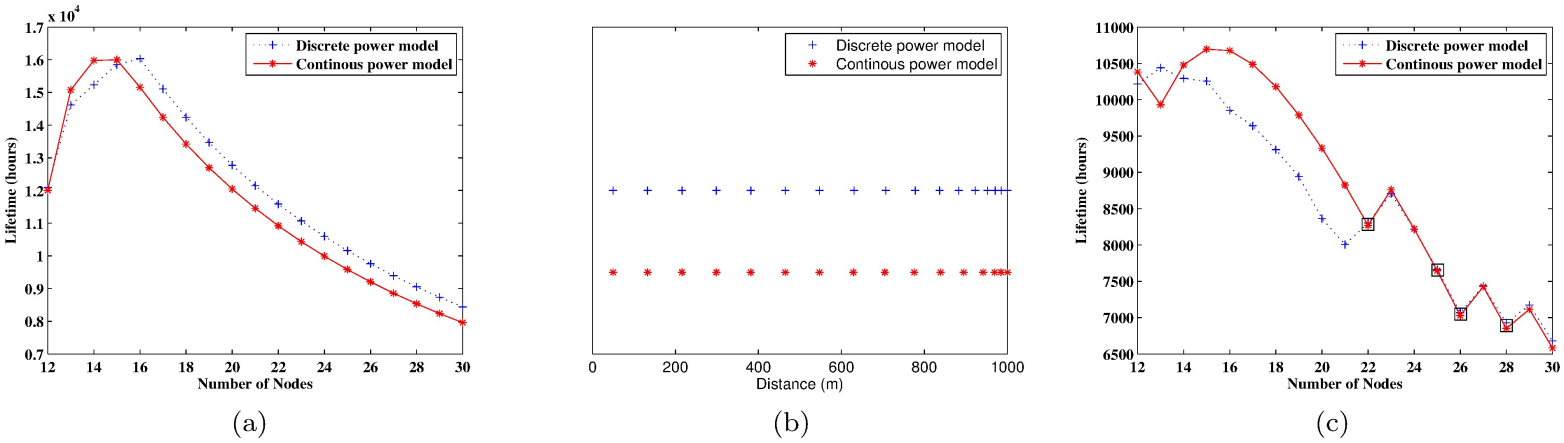
The case study results of a one-tiered WSN. (a) the optimal lifetime of a non-uniformly deployed WSN with different numbers of SNs; (b) the optimal node deployment of a non-uniformly deployed WSN; (c) the lifetime of a uniformly deployed WSN with different numbers of SNs.

Using this optimization model, we can find the optimal lifetime of the WSN under certain given number of sensors, and the optimal value of *n*_*h*_ is obtained at 16 (see the blue dotted line in Fig 4(a)). The optimal lifetime of the network does not always increase along with the number of sensors, because the reduced energy consumption from a decrease in transmission distance is offset by the increased energy consumption from transmitting the additional sensing data monitored by excessive SNs. The blue crosses in Fig 4(b) presents the optimal node deployment. To avoid the energy-hole problem, the distances between adjacent SNs are smaller for the nodes closer to the BS.

As the importance of considering the retransmission in the lifetime optimization problem has been verified in our previous studies (see [35, 36]). Here, we focus on comparing the optimal lifetimes using our model that considering the discrete power control and the ones under the continuous power model. Fig 4(a) shows the big difference of optimal lifetimes using discrete and continuous power models. From the red solid line, we can see that the optimal value of *n*_*h*_ is obtained at 15 with the continuous power model, and the corresponding optimal deployment is shown as red stars in Fig 4(b). One can see that the optimal results are quite different. Using the optimal node deployment obtained with the continuous power model (the red stars in Fig 4(b)), the actual network lifetime is only 14,429 hours (i.e., 601.2 days). This result is different from the optimal lifetime in Fig 4(a), as the nodes cannot truly adjust their output power continuously, and the continuous power model is substituted by the discrete one to find the actual lifetime. This actural lifetime is much shorter than the optimal lifetime of 16,031 hours (i.e., 668.0 days) obtained directly with the discrete power model. The large difference shows the importance of using an accurate power model in the deployment-based lifetime optimization.

Fig 4(c) illustrates the lifetime under uniformly deployed nodes. The optimal lifetime is obtained at *n*_*h*_ = 15 with the continuous power model and *n*_*h*_ = 13 with the discrete power model. However, if the optimal uniform deployment scheme obtained with the continuous power model is used for a WSN whose nodes actually use a discrete power model, the network lifetime is only 10,256 hours—181 hours shorter than the optimal lifetime obtained based on the discrete power model itself. Moreover, the WSN lifetime can be noticeably improved using the non-uniform deployment scheme. The optimal lifetime of a WSN with uniformly deployed nodes is 10,438 hours; however, the lifetime can be increased to 16,031 hours using the non-uniform deployment scheme (i.e., the network can live 7.8 months longer under the non-uniform deployment). Note that a black rectangle in Fig 4(c) means that the deployment under such number of nodes cannot satisfy the constraint of the transmission success rate. In this figure, the lifetime of a uniformly deployed WSN is not a continuous curve, because the power consumed under different transmission distances also has a stepwise function.

### Two-tiered example

In this example, MICA2 motes with CC1000 chips are used as SNs, and CC1101 chips are used as the radio modules of the RNs. In a two-tiered linear WSN, an SN transmits sensing data to its RN hop by hop; the sensing data is aggregated at the RNs into one (i.e., of *m*_*sd*_ data), and the aggregated sensing data is then relayed to the BS by the RNs hop by hop. Because CC1101 chips use binary FSK modulation and are encoded by Manchester code, the retransmission rates for RNs can be calculated using Eq (1). As with the case presented in the one tiered example, the MAC protocol of the RNs adopts the CSMA/CA protocol without RTS/CTS and the zero-idle protocol. The transmission power and range for the CC1101 chip are listed in Table 5, and other RN parameters are shown in Table 6. The values of *β*_1_, *β*_2_, *β*_3_ and *β*_4_ were obtained using curve fitting with 95% confidence bounds.

**Table 5:** Transmission power and range for CC1101 (*f* = 868 MHz, *α* = 3.95).

*P*_*o*_ (dBm)	*P*_*t*_ (mW)	*R*_max_ (m)	*P*_*o*_ (dBm)	*P*_*t*_ (mW)	*R*_max_ (m)
-30	36.3	32.69	0	50.7	187.90
-20	38.1	58.56	5	63.0	251.49
-15	40.2	78.37	7	80.4	282.59
-10	45.0	104.90	10	97.2	336.59

**Table 6:** Parameters for RNs.

Parameters	Value	Parameters	Value	Parameters	Value
*R*_*t*_	336.59 m	*P*_*r*_	46.2 mW	*β*_1_	34.38 *μ*J/bit
*E*(0)	54 kJ	*P*_*id*_	5.1 mW	*β*_2_	0.00688 pJ/bit/m^3.95^
*r*	1.2 kbit/s	*B*_*N*_	96.4 kHz	*β*_3_	38.5 *μ*J/bit
n*	40	*P*_*n*_	-121 dB	*β*_4_	4.25 *μ*J/bit
*α*	3.95	*PL*(1)	31 dB		

According to the optimization model in the Method section, the optimization model in this case can be written as

$$\begin{array}{c} \max\;L=\min_{\text{all}\;i,j}[\frac{E_{h}\left(0\right)}{E_{i}},\frac{E_{l}\left(0\right)}{E_{i,j}}]t, \end{array}$$ (24)

subject to

$$\begin{array}{c} \begin{array}{c} \frac{CovL_{1,0}+\sum_{i=1}^{n_{h}+1}\sum_{j=1}^{n_{i}}CovL_{i,j}}{D}\geq Cov_{wsn}^{*},\\ \frac{Con_{n_{h}+1}+\max_{i=1}^{n_{h}}(\sum_{k=i}^{n_{h}}Con_{k}\prod_{k=i}^{n_{h}}\Psi_{k})}{n_{l}}\geq Con_{wsn}^{*},\\ \frac{Suc_{n_{h}+1}+\sum_{i=1}^{n_{h}}(\prod_{k=i}^{n_{h}}S_{k}\times Suc_{i})}{\sum_{i=1}^{n_{h}+1}\sum_{j=1}^{n_{i}}m_{sd,i,j}}\geq Suc_{wsn}^{*},\\ n_{h}\leq n_{h}^{*},\\ n_{l}\leq n_{l}^{*},\\ \sum_{j=0}^{n_{i}}d_{i,j}=d_{i-1},\;\;\;\left(1\leq i\leq n_{h}+1\right),\\ \sum_{i=0}^{n_{h}}d_{i}=D. \end{array} \end{array}$$ (25)

Similarly, when $Cov_{wsn}^{*}=1$, $Con_{wsn}^{*}=1$ and all *m*_*sd*_(*i*) = *m*_*sd*_, Eq (25) can be simplified to

$$\begin{array}{c} \begin{array}{c} d_{i,j}\leq R_{S},\;\left(i=1,j=0\;\text{or}\;i=n_{h}+1,j=n_{n_{h}+1}\right),\\ d_{i,n_{i}}+d_{i+1,0}\leq 2R_{S},\;\left(1\leq i\leq n_{h}\right),\\ d_{i,j}\leq 2R_{S},\;\left(j\neq 0\;\text{and}\;j\neq n_{i}\right)\;,\\ d_{i,j}\leq R_{t,l},\;\left(1\leq i\leq n_{h}+1\;\text{and}\;1\leq j\leq n_{i}\right),\\ d_{i}\leq R_{t,h},\;\;\left(1\leq i\leq n_{h}\right),\\ \frac{Suc_{n_{h}+1}+\sum_{i=1}^{n_{h}}(\prod_{k=i}^{n_{h}}S_{k}\times Suc_{i})}{\sum_{i=1}^{n_{h}+1}\sum_{j=1}^{n_{i}}m_{sd,i,j}}\geq Suc_{wsn}^{*},\\ n_{h}\leq n_{h}^{*},\\ n_{l}\leq n_{l}^{*},\\ \sum_{j=0}^{n_{i}}d_{i,j}=d_{i-1},\;\;\;\left(1\leq i\leq n_{h}+1\right),\\ \sum_{i=0}^{n_{h}}d_{i}=D. \end{array} \end{array}$$ (26)

**Fig 5:**
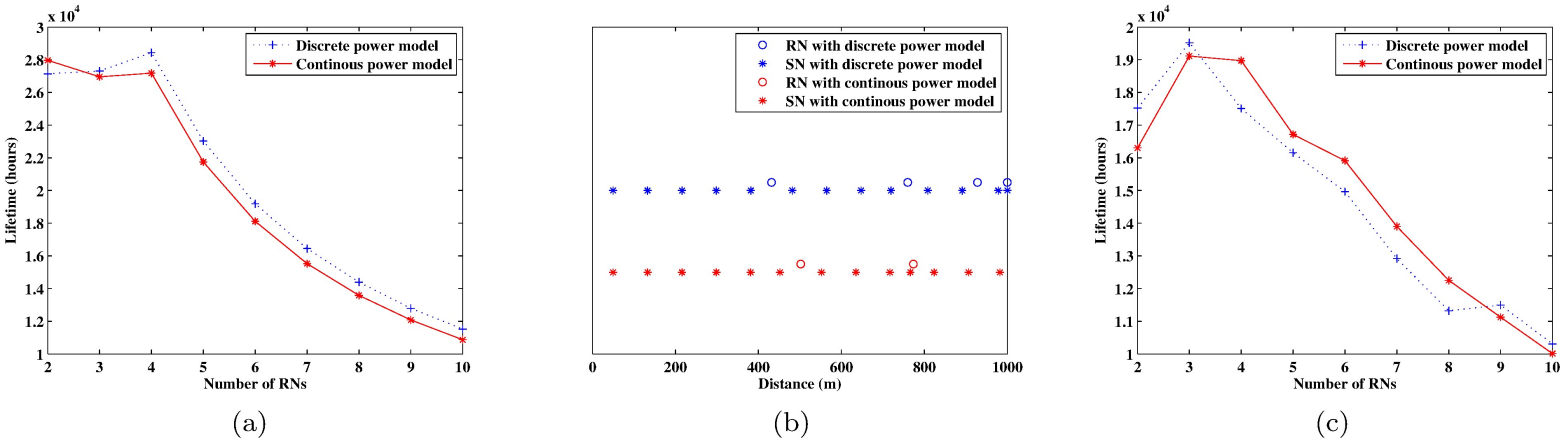
The case study results of a two-tired WSN. (a) the optimal lifetime of a non-uniformly deployed WSN with different numbers of RNs; (b) the optimal node deployment of a non-uniformly deployed WSN; and (c) the lifetime of a uniformly deployed WSN with different numbers of RNs.

Based on the optimization model above, the optimal network lifetime with different numbers of RNs is illustrated in Fig 5(a). The optimal values for *n*_*h*_ and *n*_*l*_ are obtained at 4 and 13 with the discrete power model and at 2 and 13 with the continuous one, respectively. The optimal node deployments are illustrated in Fig 5(b). Similar to the one-tiered WSN, those nodes closer to the BS and/or RN are separated by smaller distances. If the optimal deployment scheme obtained with the continuous power model is used a WSN with nodes that truly use a discrete power model, the lifetime of the WSN will be 3,708.19 hours (i.e., 154.5 days) less than the optimal lifetime obtained by using the discrete model directly. In contrast, the optimal lifetime of the two-tiered WSN is 28,427.76 hours using the discrete power model, extending the optimal lifetime of the one-tiered network by 12,396.3 hours. This result clearly shows the effectiveness of using RNs to relay the sensing data.

Fig 5(c) illustrates the lifetime under the uniformly deployed nodes. The optimal lifetime is obtained at *n*_*h*_ = 3 and *n*_*l*_ = 16 using either a discrete or continuous power model. However, one still can see that the two optimal curves under different RNs are quite different. For example, using the continuous power model, the optimal lifetime with 4 RNs is much longer than the one with 2 RNs, however, in reality, using the discrete power model, the two lifetimes are quite close.

## Conclusions

In this study, both retransmission and discrete power control, which largely influence the lifetime of the WSN but usually ignored and substituted by the continues power model respectively, are considered in the deployment based network lifetime optimization problem. To maximize the lifetime of both one-tiered and two-tiered linear WSNs, optimization models are provided with considerations of coverage, connectivity and transmission success rate. The case study verifies the importance of considering retransmission and discrete transmit power levels in the deployment based lifetime optimization problem.

The contributions of our paper include the followings: (1) the energy consumption based lifetime model is developed using the discrete power model while considering the need for data retransmissions for linear WSNs, making the lifetime evaluation more realistic; (2) the constraints of the node deployment, including the coverage, the connectivity and the transmission success probability, are analyzed for both one tiered and two tiered linear WSNs, and generic computation models are provided for these three constraints; and (3) an optimization model is developed for the deployment based lifetime problem using the more realistic network lifetime evaluation method, and the optimal result provides a longer lifetime in practice.

This paper focused on linear WSNs. In a future extension, we plan to study deployment-based lifetime optimization for WSNs with planar and hierarchical structures.

## Supporting information

S1 Appendix Number of transmission attempts and transmission success rate. (PDF)

S2 Appendix Continuous energy consumption model. (PDF)
