# Supplementary material for: Deployment-based lifetime optimization for linear wireless sensor networks considering both retransmission and discrete power control
Source: PLoS One. 2017 Nov 29;12(11):e0188519. doi: 10.1371/journal.pone.0188519 (PMC5706707; doi:10.1371/journal.pone.0188519)
Supplement: S1 Appendix — (PDF) [file pone.0188519.s001.pdf]

Appendix 1: Number of transmission attempts and transmission success rate As in [1], there are three possible situations for any single transmission attempt: (1) both the sensing data and the ACK response are transmitted successfully; (2) the sensing data is transmitted successfully, but the ACK response fails; and (3) the sensing data transmission fails; therefore, no ACK response is sent to the transmitter. When the transmitter fails to receive an ACK response, it will retransmit the sensing data until the maximum retry threshold is reached. Table 1 lists all 15 possibilities of both transmission and reception processes when sending one sensing data transmission between two hops with the maximum retry threshold as 2.

Table 1: **Sensing data transmission and reception possibilities between two hops.**

Transmission attempts						Number of attempts		Probability	Success or fail
1#		2#		3#					
SD	ACK	SD	ACK	SD	ACK	SD	ACK		
1	1	N/A	N/A	N/A	N/A	1	1	$(1-A)(1-B)$	S
1	0	1	1	N/A	N/A	2	2	$B(1-A)^2(1-B)$	S
0	N/A	1	1	N/A	N/A	2	1	$A(1-A)(1-B)$	S
1	0	1	0	1	1	3	3	$B^2(1-A)^3(1-B)$	S
1	0	1	0	1	0	3	3	$B^3(1-A)^3$	S
1	0	1	0	0	N/A	3	2	$AB^2(1-A)^2$	F
1	0	0	N/A	1	1	3	2	$AB(1-A)^2(1-B)$	S
1	0	0	N/A	1	0	3	2	$AB^2(1-A)^2$	S
1	0	0	N/A	0	N/A	3	1	$A^2B(1-A)$	F
0	N/A	1	0	1	1	3	2	$AB(1-A)^2(1-B)$	S
0	N/A	1	0	1	0	3	2	$AB^2(1-A)^2$	S
0	N/A	1	0	0	N/A	3	1	$A^2B(1-A)$	F
0	N/A	0	N/A	1	1	3	1	$A^2(1-A)(1-B)$	S
0	N/A	0	N/A	1	0	3	1	$A^2B(1-A)$	S
0	N/A	0	N/A	0	N/A	3	0	A^3	F

Notes: (1) In Columns 1 $\sim$ 6, SD denotes sensing data, and 1, 0 and N/A represent transmission success, failure, and no transmission, respectively. (2) In Column 9, A and B are the retransmission rates of the sensing data and the ACK, respectively, which can be calculated using Eq. (4). (3) In Column 10, S and F denote the success (S) or failure (F), respectively, of sensing data transmission when the maximum retry threshold is set to 2.

According to Table 1, the probabilities of transmitting the sensing data and ACK k times at Node N_y with transmission distance d_y can be calculated as follows:

$$\omega_{sd,k}(d_y) = \begin{cases} (1-A)(1-B), & k=1, \\ (1-A)(1-B)(A+B-AB), & k=2, \\ A^2+2AB(1-A)+B^2(1-A)^2, & k=3, \end{cases} \tag{1}$$

and

$$\omega_{ack,k}(d_y) = \begin{cases} (1-A^2)(1-B)+A^2(1-A)(1+2B), & k=1, \\ B(1-A)^2(1-B)+AB(1-A)^2(2+B), & k=2, \\ B^2(1-A)^3, & k=3. \end{cases} \tag{2}$$

respectively. Then, the expected number of transmission attempts for the two types of data can be calculated by

$$\begin{cases} \omega_{sd}(d_y)=\omega_{sd,1}(d_y)+2\omega_{sd,2}(d_y)+3\omega_{sd,3}(d_y), \\ \omega_{ack}(d_y)=\omega_{ack,1}(d_y)+2\omega_{ack,2}(d_y)+3\omega_{ack,3}(d_y). \end{cases} \tag{3}$$

Moreover, the successful transmission rate of one sensing data sent between two adjacent hops can be denoted by

$$S_y = 1 - A^3 - 2A^2B\,(1-A) - AB^2(1-A)^2. \tag{4}$$

References

[1] Li R, Liao H, Liu X, Huang N. Lifetime optimisation for linear wireless sensor networks under retransmission. International Journal of Ad Hoc and Ubiquitous Computing. 2016;22(3):153–163.
