# Supplementary material for: Deployment-based lifetime optimization for linear wireless sensor networks considering both retransmission and discrete power control
Source: PLoS One. 2017 Nov 29;12(11):e0188519. doi: 10.1371/journal.pone.0188519 (PMC5706707; doi:10.1371/journal.pone.0188519)
Supplement: S2 Appendix — (PDF) [file pone.0188519.s002.pdf]

S2 Appendix. Continuous energy consumption model. We consider three types of energy consumption: data transmission, data reception and idle state. According to the most widely used continuous energy consumption model proposed by Heinzelman et al. [1], the continuous energy consumption at Node N_y in Layer x during the data-gathering cycle can be written as follows:

$$\begin{aligned} E_y &= E_{t,y} + E_{r,y} + E_{id,y} \\ &= (\beta_1 + \beta_2 d^{\alpha}) m_{t,y} + \beta_3 m_{r,y} + \beta_4 t_{id,y} r_x, \end{aligned} \tag{1}$$

where β_1, β_2, β_3 and β_4 are system parameters that represent the amount of energy consumed per bit in the transmission circuitry, transmission amplifier, and receiving circuitry, respectively, and β_4 represents the amount of energy consumed per bit duration in the idle state. According to [1], the typical values are $\beta_1 = \beta_3 = 50nJ/bit$ and $\beta_2 = 100pJ/bit/m^2$.

References

[1] Heinzelman WR, Chandrakasan A, , Balakrishnan H. Energy-efficient communication protocol for wireless microsensor networks. In: Proceedings of the 33rd Annual Hawaii International Conference on System Sciences. Hawaii, USA; 2000. p. 1–10.
